# Vertical-guided bone regeneration with a titanium-reinforced d-PTFE membrane utilizing a novel split-thickness flap design: a prospective case series

**DOI:** 10.1007/s00784-020-03617-6

**Published:** 2020-10-10

**Authors:** Peter Windisch, Kristof Orban, Giovanni E. Salvi, Anton Sculean, Balint Molnar

**Affiliations:** 1 Faculty of Dentistry, Department of Periodontology, Semmelweis University, Budapest, Hungary; 2 Department of Periodontology, School of Dental Medicine, University of Bern, Freiburgstrasse 7, 3010 Bern, Switzerland

**Keywords:** Guided bone regeneration, Vertical augmentation, Split-thickness flap, Implant placement, Non-resorbable membrane, Autogenous bone, Xenograft

## Abstract

**Objectives:**

To evaluate the feasibility of a newly proposed minimally invasive split-thickness flap design without vertical-releasing incisions for vertical bone regeneration performed in either a simultaneous or staged approach and to analyze the prevalence of adverse events during postoperative healing.

**Materials and methods:**

Following preparation of a split-thickness flap and bilaminar elevation of the mucosa and underlying periosteum, the alveolar bone was exposed over the defects, vertical GBR was performed by means of a titanium-reinforced high-density polytetrafluoroethylene membrane combined with particulated autogenous bone (AP) and bovine-derived xenograft (BDX) in 1:1 ratio. At 9 months after reconstructive surgery, vertical and horizontal hard tissue gain was evaluated based on clinical and radiographic examination.

**Results:**

Twenty-four vertical alveolar ridge defects in 19 patients were treated with vertical GBR. In case of 6 surgical sites, implant placement was performed at the time of the GBR (simultaneous group); in the remaining 18 surgical, sites implant placement was performed 9 months after the ridge augmentation (staged group). After uneventful healing in 23 cases, hard tissue fill was detected in each site. Direct clinical measurements confirmed vertical and horizontal hard tissue gain averaging 3.2 ± 1.9 mm and 6.5 ± 0.5 mm respectively, in the simultaneous group and 4.5 ± 2.2 mm and 8.7 ± 2.3 mm respectively, in the staged group. Additional radiographic evaluation based on CBCT data sets in the staged group revealed mean vertical and horizontal hard tissue fill of 4.2 ± 2.0 mm and 8.5 ± 2.4 mm. Radiographic volume gain was 1.1 ± 0.4 cm^3^.

**Conclusion:**

Vertical GBR consisting of a split-thickness flap and using titanium-reinforced non-resorbable membrane in conjunction with a 1:1 mixture of AP+BDX may lead to a predictable vertical and horizontal hard tissue reconstruction.

**Clinical relevance:**

The used split-thickness flap design may represent a valuable approach to increase the success rate of vertical GBR, resulting in predicable hard tissue regeneration, and favorable wound healing with low rate of membrane exposure.

**Electronic supplementary material:**

The online version of this article (10.1007/s00784-020-03617-6) contains supplementary material, which is available to authorized users.

## Introduction

During the past decades, dental implant therapy has become a frequently chosen solution to replace missing teeth. In order to achieve long-term success and esthetic results, optimal amounts of vertical and horizontal hard tissue dimension as well as an adequate soft tissue environment are required. The three-dimensional resorption of the alveolar ridge is one of the most unwanted biological processes following tooth extraction; resorption is more progressive in patients with periodontal disease due to unfavorable hard and soft tissue conditions [1–3]. As a result, edentulous sites often present compromised dimensions, and therefore, ridge augmentation may be required before or at the time of implant placement.

Several reconstructive surgical methods are suggested in literature to rebuild the deficient alveolar ridge. Transplantation of autogenous bone blocks (AB) is a well-documented surgical approach to reconstruct three-dimensional alveolar defects. This technique requires a relatively moderate healing time of 4–6 months, but the resorption rate of AB as well as the quality and survival of transplanted tissues shows high individual variations [4–6]. The most frequently reported surgical technique to rebuild missing alveolar bone is guided bone regeneration (GBR), which has been shown to deliver predictable long-term results in terms of crestal bone stability, implying however a longer healing period (i.e., 6–9 months) [7–9]. By using a barrier membrane, a secluded space should be created to prevent epithelial migration into the wound and while the particulated auto- or xenografts may stabilize the blood clot and facilitate bone formation [10]. The GBR technique is feasible for both horizontal and vertical reconstruction of edentulous sites utilizing resorbable or non-resorbable membranes [6, 11–13].

Vertical ridge augmentation yields less predictable treatment outcomes compared with horizontal ridge augmentation due to the fact that it requires advanced flap management and uncompromised soft tissue coverage of the wound to protect the grafts and to support supracrestal blood clot stabilization [14]. It has been demonstrated that the blood and cell supply during the healing process following ridge reconstructions mainly originates from the periosteum [15]. The elevation of the periosteum in order to support both revascularization and tissue integration of supracrestally positioned grafting materials is not only surgically extremely challenging but also implies a longer healing time for the regeneration process. Numerous studies have demonstrated the advantages of reinforced non-resorbable membranes to achieve successful vertical hard tissue reconstruction by maintaining and protecting the space for the blood clot and the filler material and by excluding soft tissue penetration [16].

Non-resorbable expanded polytetrafluoroethylene (e-PTFE) membranes are accepted as the gold standard for vertical GBR due to their favorable mechanical and barrier properties. Since the main rationale for GBR membrane rigidity is to ensure space maintenance, the most commonly used e-PTFE membranes are reinforced by titanium. In this way, the risk for graft compression during the healing period can be reduced over the biologically determined 9 months of healing required for graft maturation and corticalization.

Early wound dehiscence and membrane exposure are the main reasons for decreased treatment predictability following the application of non-resorbable membranes during GBR procedures. Bacterial colonization of exposed membrane surfaces inevitably leads to tissue inflammation and graft disintegration requiring premature membrane removal before the completion of the healing period, which leads to reduced hard tissue formation [17].

If properly executed, the conventional full-thickness flap design with vertical and horizontal releasing incisions results in tension-free wound closure and subsequent primary intention wound healing; however, insufficient healing and membrane exposure is a well-documented complication of this approach [18]. Apart from decreasing flap tension, the placement of incisions, which disrupt the continuity of the periosteal layer, may negatively affect periosteal blood supply. As an alternative to the classical full-thickness flap design for vertical GBR, a split-thickness flap approach was suggested by several authors, which might similarly result in a tension-free wound closure but at the same time avoiding the previously mentioned adverse events related to full-thickness flaps [19, 20]. More recently, in order to additionally improve the healing and to improve the predictability of vertical augmentation procedures, our group proposed a novel minimally invasive split-thickness flap design without vertical-releasing incisions [13].

Particulated autogenous bone grafts (AP) have highly active biological capacity and are preferred based on their osteoinductive properties; nevertheless, they are prone to early resorption when used alone for GBR [16]. Therefore, in order to prolong graft stability and to minimize the amount of harvested autogenous bone, recent studies have suggested to combine AP with bovine-derived xenograft (BDX). The efficacy of a mixed 1:1 ratio of AP + BDX has been reported by several authors [21–24]. BDX is the most commonly used and researched xenogeneic material for GBR with proven osteoconductive effect and volumetric stability [25].

According to the literature, when applying non-resorbable membranes in combination with AP + BDX for vertical GBR, approximately 9 months of healing is needed for proper graft maturation and tissue integration, allowing for implant placement and long-term crestal bone maintenance. In cases of minor vertical alveolar defects with mild to moderate hard tissue loss, primary stability of a dental implant in standard length and width can be achieved. In such cases, a GBR approach can be used simultaneously to implant placement [26]. If the abovementioned prerequisites for implant placement are not given, the staged approach is indicated, implying ridge reconstruction 9 months prior to implant placement [22].

However, at present, the data on the outcomes of vertical bone augmentation by means of GBR are still scarce and controversy exists on the predictability of these approaches. However, at present, it is unknown to what extent our recently described minimally invasive split-thickness flap design without vertical-releasing incisions [13] may lead to predictable clinical outcomes in terms of postoperative complications and treatment outcomes. Therefore, the aims of the present prospective case series study were (**a**) to evaluate the feasibility of the proposed minimally invasive split-thickness flap design without vertical-releasing incisions for vertical bone regeneration performed in either a simultaneous or staged approach and (**b**) to record and analyze the prevalence of adverse events during postoperative healing.

## Materials and methods

The present case series was performed in patients with advanced chronic periodontitis (grade III, stage B) [27] presenting localized three-dimensional alveolar ridge defects requiring surgical bone augmentation to allow implant placement. Patients underwent comprehensive periodontal treatment prior to surgery, no residual pockets deeper than 3 mm were present, full-mouth plaque score were less than 20%, and full-mouth bleeding scores were less than 15%. Teeth were extracted at least 6 months prior to surgery. The reasons for tooth loss were either severe attachment and bone loss (i.e., attachment and bone loss reaching the apex of the teeth and/or class III furcation involvements) or complicated perio-endodontic lesions.

All patients were selected and treated at the Department of Periodontology, Semmelweis University, Budapest, Hungary, between January 2012 and June 2015.

The study protocol was approved by the Semmelweis University Regional and Institutional Committee of Science and Research Ethics (Approval Number 77/2011). Surgical interventions were undertaken with the understanding and written informed consent of each subject. The patients were treated in full accordance with ethical principles, including the World Medical Association Declaration of Helsinki (version 2008).

### Preoperative care

Preoperatively, supra- and subgingival scaling was performed, patients received individual oral hygiene instructions and maintained a high level of oral hygiene throughout the whole treatment period (full-mouth plaque score and full-mouth bleeding score did not exceed 25%). Presurgical patients used chlorhexidine digluconate 0.2% mouthrinse (Curasept ADS 220, Curaden AG, Kriens, Switzerland) for 2 min.

### Surgical technique

The surgical approach was described in detail elsewhere [13]. Briefly, in both groups’ local anesthesia (4% articaine-hidrocloride with 0.0001% epinephrine - Ultracain DS Forte, Sanofi-Aventis, Paris, France), a midcrestal incision was placed on the keratinized mucosa with no. 15 blades (Aesculap, Braun AG, Tuttlingen, Germany). The midcrestal incision was continued intracrevicularly at the two adjacent teeth mesially and distally both buccally and orally with no. 15C blades. In case of posterior edentulism, the midcrestal incision line length was two-thirds of the entire surgical area, and one-third length was continued mesially to the neighboring two teeth. No vertical-releasing incisions were performed. A full-thickness buccal flap was reflected with elevators up to the MJ, followed by split-thickness mucosal flap preparation over the mucogingival line (MJ). Subsequently, the underlying periosteal layer was elevated from the bone surface. In the simultaneous group, 3.3-mm or 4.1-mm diameter bone level implants (Straumann AG, Basel, Switzerland) were placed in a prosthetically predefined position using surgical guides. No implants with 3.3-mm diameter were placed in molar positions. All implants were positioned supracrestally according to their preplanned prosthetic positions. A single-use disposable bone scraper (Safescraper, Osteogenics Biomedical, Lubbock, TX, USA) was used to harvest AP from the lateral surface of the adjacent alveolar ridge. Bone chips were mixed with BDX (Bio-Oss, Geistlich AG, Wolhusen, Switzerland) in a 1:1 ratio. The 1:1 mixture of AP + BDX was placed laterally and supracrestally to the alveolar ridge. A non-resorbable high-density PTFE (d-PTFE) membrane (Cytoplast, Osteogenics Biomedical, Lubbock, USA) was fixed with titanium pins (Frios Membrane Tacks, Dentsply, York, USA). Double-layer suturing was performed using 4-0 horizontal mattress sutures (Supramid, Braun AG, Tuttlingen, Germany) in order to cover the membrane with the periosteal layer, while 5-0 horizontal mattress and non-interrupted sutures (Supramid, Braun AG, Tuttlingen, Germany) were utilized to close the mucosal layer and to reach a tension-free wound closure. Sutures were removed after 14 days.

**Fig. 1:**
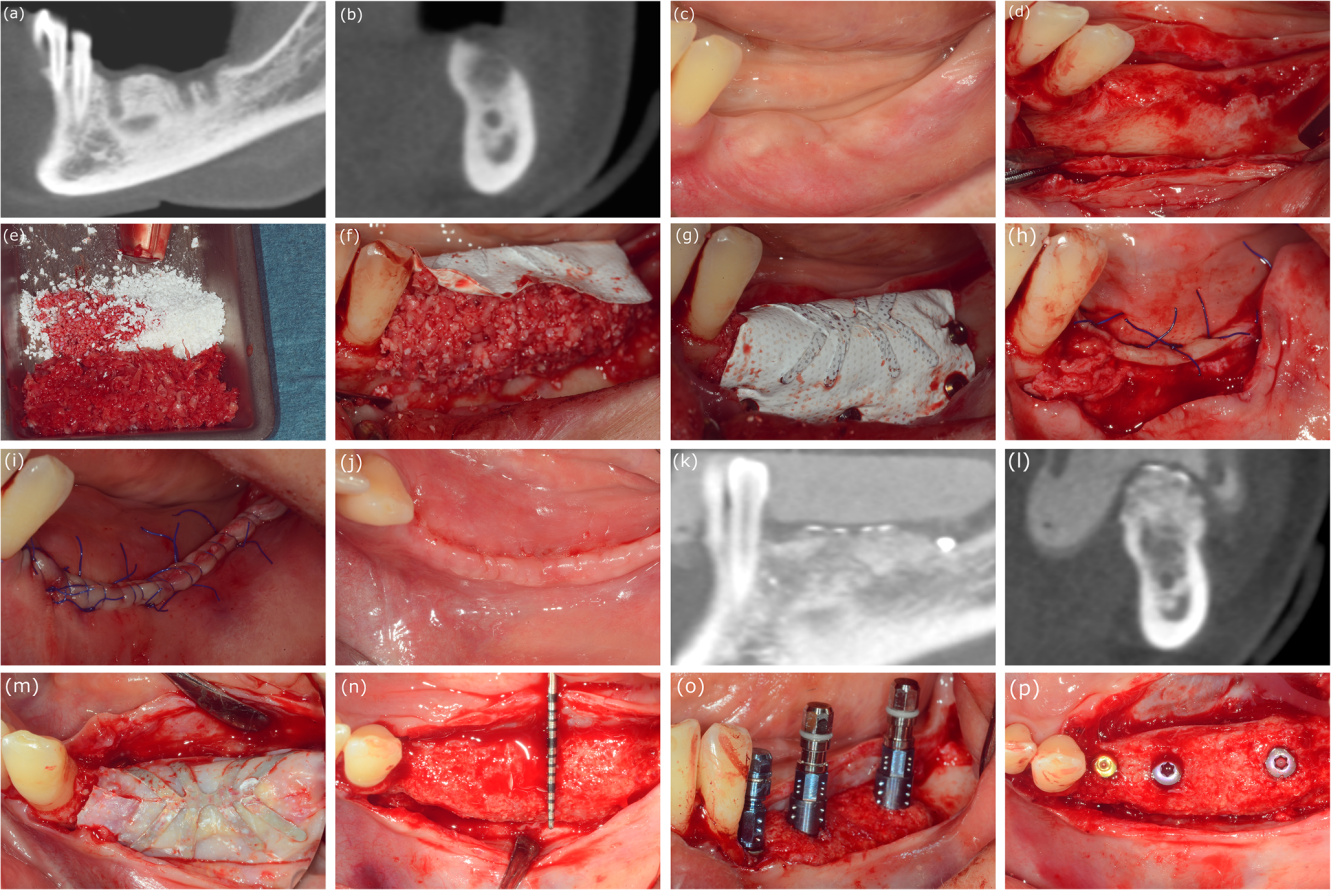
Case presentation of patient no. 11, (case no. 13 - left mandible) from the staged group. **a** Baseline CBCT scan, parasagittal section: 2 months after tooth extraction, horizontovertical alveolar defect. **b** Baseline CBCT scan, frontal section: 2 months after tooth extraction, horizontovertical alveolar defect. **c** Clinical view of edentulous mandible. **d** Split-thickness flap preparation. **e** Bovine-derived xenograft (Geistlich Bio-Oss) and particulate autogenous bone graft. **f** Adaptation of non-resorbable membrane (Osteogenics Cytoplast) over the composite graft. **g** Membrane fixation using titanium pins. **h** Double-layer suturing: tension-free periosteal layer closure with horizontal mattress sutures. **i** Mucosal layer closure with horizontal mattress and non-interrupted sutures. **j** Wound healing 2 weeks after surgery. **k** Postoperative CBCT scan: 9 months after GBR parasagittal section: vertical bone gain. **l** Postoperative CBCT scan: 9 months after GBR, frontal section: vertical and horizontal bone gain. **m** Membrane removal at 9 months reentry. **n** Optimal amount of hard tissue for implant placement. **o** Guided implant placement (Straumann bone level). **p** Prosthetically driven implant positioning

In the staged group, at 9 months, a split-thickness flap was elevated in the same way as described above. The titanium pins and the d-PTFE membrane were removed, and 3.3-mm or 4.1-mm diameter bone level implants (Straumann AG, Basel, Switzerland) were placed in a prosthetically predefined position using a surgical template (Fig. 1a–p). Also, in this group, no implants with 3.3-mm diameter were placed in molar positions. The flap was closed with horizontal mattress sutures and single interrupted sutures. Soft tissue augmentation was performed at the time of implant placement if the vertical dimension of the soft tissue thickness was less than 2 mm over the inserted implants. Two months later, in cases where less than 3-mm width of keratinized mucosa was present, palatal epithelialized free gingival grafts were harvested and placed. After another 2 months, implant uncovery was performed.

**Fig. 2:**
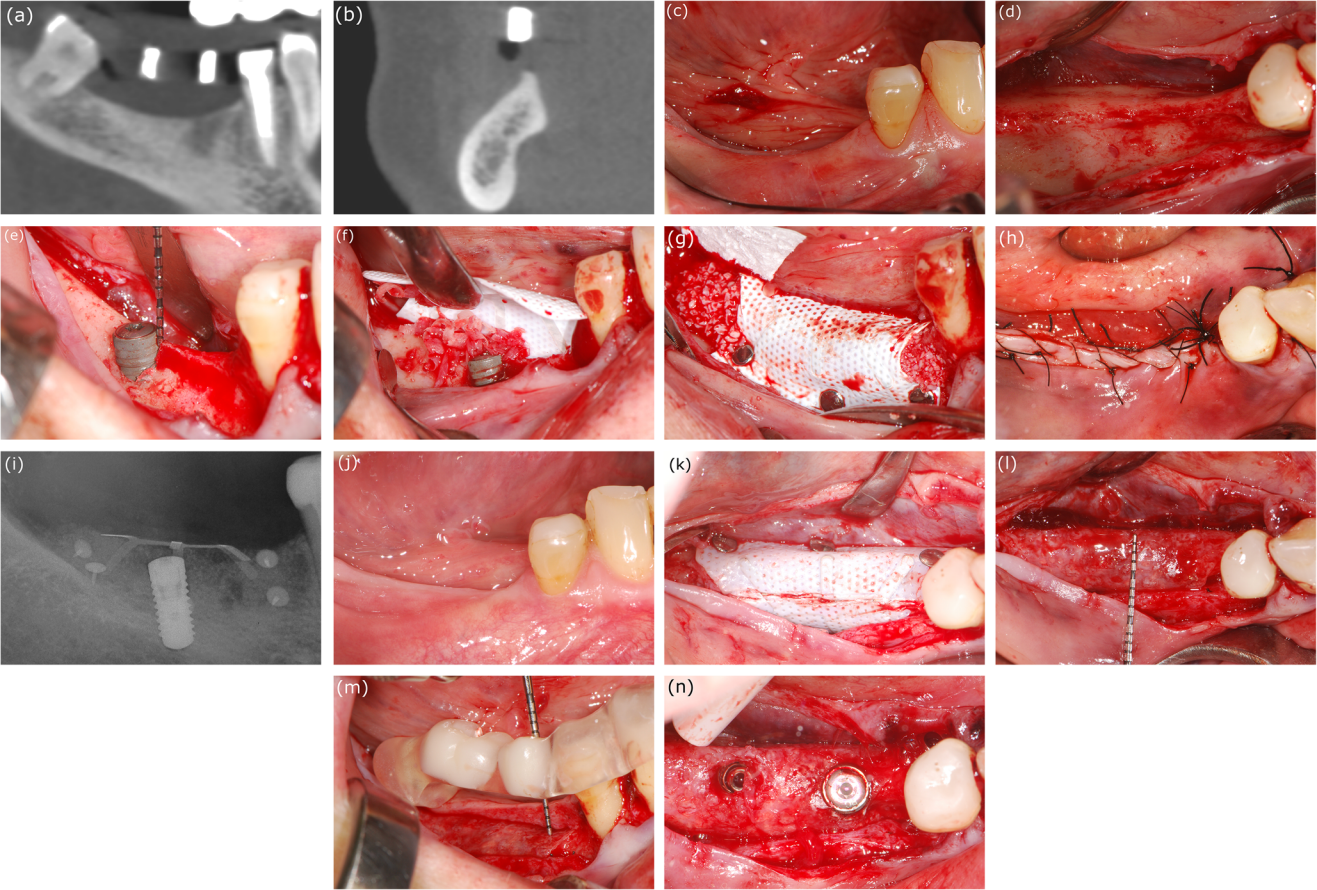
Case presentation of patient no. 4 (case no. 5 - right mandible) from the simultaneous group. **a** Baseline CBCT scan, parasagittal section: horizontovertical alveolar defect. **b** Baseline CBCT scan, frontal section: horizontovertical alveolar defect. **c** Clinical view of edentulous mandible. **d** Split-thickness flap preparation. **e** Guided implant placement, supracrestally positioned implant (Straumann bone level). **f** Adaptation of non-resorbable membrane (Osteogenics Cytoplast) over the composite graft. **g** Membrane fixation using titanium pins. **h** Double-layer suturing. **i** Postoperative intraoral X-ray. **j** Healed alveolar ridge 9 months after simultaneous GBR. **k** Membrane removal at 9 months reentry. **l**, **m**, **n** Optimal amount of hard tissue; previously placed distal implant is covered with hard tissue, additional implant placed mesially

In the simultaneous group, membranes and titanium pins were removed at 9 months after the first surgery, and the implants were only uncovered if an adequate peri-implant soft tissue width (i.e., at least 3 mm) surrounding the implants was present (Fig. 2 a–n). Soft tissue augmentation was performed at the time of membrane removal if the peri-implant soft tissue thickness (vertical dimension) was less than 2 mm. The above described reconstruction of keratinized tissues was performed 2 months before the second-stage surgery only in cases without an adequate width and thickness or absence of keratinized tissue.

### Postoperative care

Postoperatively, antibiotic therapy (Penicillin with Clavulanic acid 2 × 1000 mg/day; Augmentin Duo, GlaxoSmithKline, Brentford, UK) and non-steroidal anti-inflammatory drugs (Diclofenac-Sodium 4 × 50 mg/day; Cataflam, Novartis International AG, Basel, Switzerland) were prescribed for 1 week in order to avoid infections and to decrease swelling and pain. In case of penicillin allergy, Clindamycin (Dalacin C, Pfizer, New York, USA) 4 × 300 mg per day was prescribed. Patients were instructed to gently brush teeth at surgical sites with a soft manual toothbrush. For chemical plaque control, 0.2% chlorhexidine digluconate mouthwash (Curasept ADS 220, Curaden AG, Kriens, Switzerland) was prescribed twice a day. Mucosal sutures were removed 7 days, periosteal sutures 14 days after surgery. Following suture removal, patients were scheduled for recall visits weekly in the first month, followed by visits every 3 months postoperatively. Patients received fixed partial dentures 2 weeks after implant uncovering. After delivery of the final prosthetic reconstructions, patients were enrolled in a periodontal maintenance program.

### Clinical evaluation

Measurements were taken by a single investigator, KO, following examiner calibration. In the simultaneous group, direct measurements were performed by UNC-15 probes (HU-Friedy, Chicago, IL, USA) during the first surgery. The distance between crestal bone and the most coronal part of the supracrestally positioned implants (Clinical Vertical Dimension - c-VD) was recorded as a negative value (lack of hard tissue). Extent c-VD was recorded at 4 aspects of each implant (vestibular, oral, mesial, distal); mean values were calculated. The orovestibular dimension of the ridge at the level of the most coronal part of the implant (Clinical Horizontal Dimension - c-HD) was by definition zero. During implant recovery, c-VD and c-HD were recorded again following membrane removal; vertical and horizontal hard tissue gain was calculated (Clinical Vertical Dimension Gain - c-VDG, Clinical Horizontal Dimension Gain - c-HDG). In the staged group, both direct and radiographic evaluations were performed. c-VD and c-HD were measured directly using by UNC-15 probes and individually fabricated surgical stent during augmentation and implant placement in the planned implant position. The distance between the surgical stent and crestal bone level was recorded at the time of GBR and at implant placement. Differences were registered as c-VDG. The orovestibular dimension of the alveolar ridge at the postopoperative crestal bone level was by definition zero; thus, c-HDG calculation was based on postop orovestibular dimension at the crestal bone level.

### Radiographic evaluation

Measurements were taken by a single investigator, KO, following examiner calibration. Intraoral radiographs, orthopantomograms, and Cone Beam Tomography (CBCT) scans (i-CAT, KaVo, Biberach, Germany) were taken (120 kVp; 5 mA; 7.4 s; 0.200-mm voxel sizes; 360° rotation) to assess the three-dimensional morphology of edentulous alveolar ridges at baseline prior to surgery. Further intraoral radiographs were taken at implant loading and during recall visits on a yearly basis. Measurements were performed in the planned implant positions at every surgical site. In the staged group, a second CBCT scan was taken 9 months after GBR procedure for three-dimensional implant position planning; thus, radiographic evaluation was performed based on CBCT data (Radiographic Vertical Dimension - r-VD, Radiographic Horizontal Dimension - r-HD). For alignment of pre- and postoperative datasets, adjacent teeth were used as an anatomical reference. Radiographic Vertical Dimension Gain (r-VDG) and Radiographic Horizontal Dimension Gain (r-HDG) were calculated. If more than 1 implant was placed in the surgical area, hard tissue gain was measured in every planned implant position, and the highest value was recorded. Radiographic linear measurements were performed by the i-CAT Vision software (Imaging Sciences International, Hatfield, PA, USA). Additional 3D volumetric measurement was performed in the staged group to evaluate the hard tissue gain (Radiographic Volume - r-VOL). Patients with GBR procedure and with sinus floor elevation were excluded from 3D measurement based on augmented area separation inaccuracy. Radiographic volume measurements were performed by the Osirix software (Pixmeo Sarl, Geneva, Switzerland).

### Statistical analysis

Comparison of hard tissue changes between the two groups was not the aim of this study. “Horizontal and vertical dimension changes were recorded using the above described measurement method and therefore, only descriptive statistics were performed.”

Mean values and standard deviations (SDs) were calculated.

## Results

Systemically healthy, partially edentulous patients were treated at the Department of Periodontology, Semmelweis University, Budapest, Hungary: 24 surgical sites of 19 non-smoking subjects were selected. Treatments were performed at 8 maxillary and 16 mandibular sites. The average age was 50.3 ± 12.9 years, 2 patients were male, and 17 patients were female (Table 1.)

**Table 1:** Patient’s demographics and treatment allocation

Characteristic	No. of subjects	No. of surgical sites	No. of implants	No. of patients with volume assessment
Gender				
Male	2	2	2	0
Female	17	22	43	13
Mean age (y)	50.25 ± 12.90			
Surgical area				
Maxilla	7*	8	13	2
Mandible	13*	16	32	11
Treatment allocation				
Simultaneous group	5	6	9	0
Staged group	15	18	36	13

*One patient was presented in both groups

In the present study, 45 Straumann bone level implants were placed in 24 vertically augmented surgical sites. In the simultaneous group, implant placement was performed with simultaneous GBR (9 implants in 5 patients and 6 surgical sites) if at least 6-mm vertical bone height was detected coronally from the adjacent anatomical landmarks (floor of maxillary sinus or nasal cavity, mandibular nerve). In the staged group, implants were placed at 9 months after GBR (36 implants in 15 patients and 18 surgical sites) in cases where the residual vertical bone height was less than 6 mm. One patient was represented in both groups. Patient no. 4 in the simultaneous group is equivalent to patient no. 12 in the staged group. This patient received a dental implant in position no. 46 with simultaneous GBR, and after the healing period, the patient received another dental implant into the newly formed hard tissue in position no. 45 (Fig. 2). A total of 4 patients (i.e., 2 patients from the simultaneous and 2 patients from the staged group) displayed a single tooth gap, while the remaining patients had larger edentulous sites and received more than one implant.

All GBR procedures were successful; the healing period was uneventful in all 18 patients and 23 sites. Swelling and pain was moderate in all cases; additional medication, such as the prescription of systemic steroids, was not needed. Early membrane exposure was detected in 1 surgical site. In this particular case, the d-PTFE membrane was removed and replaced by a resorbable collagen membrane (Bio-Gide, Geistlich AG, Wolhusen, Switzerland) at 6 weeks after surgery. At the time of the reentry in the staged group, every augmented site was suitable for implant placement after membrane removal. The site with early membrane removal received additional connective tissue grafting to compensate for the loss of soft tissues.

In the simultaneous group, 5 patients with 6 surgical sites were treated, a total of 9 implants were placed simultaneously with the GBR procedure (Table 2). Mean c-VDG was 3.2 ± 1.9 mm, and mean c-HDG was 6.5 ± 0.5 mm. GBR procedure’s success rate in the simultaneous group was 92.6%, where a 100% success rate refers to implant surfaces fully covered with new hard tissue. The most coronal part of the implants was covered with newly formed hard tissue around 7 implants, representing 100% successful augmentation. Around 1 implant, implant coverage was incomplete, a mean postoperative c-VD of 2 mm (patient 1, implant in position 16) was recorded. In this case, the success rate (hard tissue gain in percentage) of the augmentation was 50%.

**Table 2:** Simultaneous group

Patient no. (site no.)	Age (y)	Sex	Arch	Mean c-VDG (mm)	Mean c-HDG (mm)
1 (1)	38	F	Maxilla	2	6.6
2 (2)	35	M	Maxilla	6.9	6.3
3 (3)	36	F	Mandible	1.75	7
4 (4)	63	F	Mandible	2.75	7
4 (5)	63	F	Mandible	2.75	6
5 (6)	42	F	Mandible	3	6
Mean				3.19	6.48
STDEV				1.88	0.46

*c-VDG* clinical vertical dimension gain, *c-HDG* clinical horizontal dimension change

In the staged group, 15 patients with 18 surgical sites were treated, a total of 36 implants were placed 9 months after GBR (Table 3). Mean c-VDG was 4.5 ± 2.2 mm, and mean c-HDG was 8.7 ± 2.3 mm.

**Table 3:** Staged group

Patient no, (site no.)	Age (y)	Sex	Arch	c-VDG (mm)	c-HDG (mm)	r-VOL (ccm)
1 (1)	69	F	Mandible	1	4	0.82
2 (2)	62	F	Maxilla	6	11	-
3 (3)	67	F	Maxilla	2	8	0.96
4 (4)	57	F	Maxilla	3	15	-
4 (5)			Maxilla	3	10	-
5 (6)	57	F	Mandible	5	8	0.89
5 (7)			Mandible	9	8	1.48
6 (8)	22	F	Maxilla	4	8	1.00
7 (9)	46	F	Mandible	6	8	0.87
8 (10)	56	F	Mandible	4	7	1.01
9 (11)	42	F	Mandible	6	10	0.60
10 (12)	43	M	Maxilla	8	7	-
11 (13)	49	F	Mandible	4	8	1.88
11 (14)			Mandible	6	9	1.81
12 (15)	64	F	Mandible	1	7	-
13 (16)	59	F	Mandible	4	10	1.49
14 (17)	38	F	Mandible	5	8	0.60
15 (18)	60	F	Mandible	4	11	0.96
Mean				4.50	8.72	1.11
STDEV				2.15	2.30	0.42

*c-VDG* clinical vertical dimension change, *c-HDG* clinical horizontal dimension change, *r-VOL* radiographic volume

Additional linear radiographic measurements in the staged group based on alignment of baseline and 9 months CBCT scans showed lower hard tissue gain compared with direct clinical measurements. Mean r-VDG was 4.2 ± 2.0 mm, while mean r-HDG was 8.5 ± 2.4 mm.

Thirteen augmented sites of 11 patients from the staged group were suitable for 3D volumetric measurement to compare the amount of the hard tissue before and 9 months after the surgery. The main r-VOL was 1.1 ± 0.4 cm^3^.

In cases which required soft tissue augmentation, the reason for the soft tissue deficiency either occured due to the baseline defect or occurred due to extensive flap mobilization and the fact that non-resorbable membranes impair periosteal blood supply of the supracrestal soft tissues. Following our protocol, the minimally required keratinized tissue thickness of 2 mm and width of 3 mm was successfully obtained in all cases.

## Discussion

The present case series has evaluated the feasibility of a newly proposed minimally invasive split-thickness flap design without vertical-releasing incisions for vertical bone regeneration performed in either a simultaneous or staged approach and also analyzed the prevalence of adverse events during postoperative healing. According to the best of our knowledge, this is the first report demonstrating predictable outcomes following vertical GBR using a split-thickness flap design.

The present material comprised a total of 24 surgical sites treated by means of vertical GBR, while hard tissue changes were assessed by clinical and 3D radiographic evaluation. Although short implants and horizontal ridge augmentation using non-resorbable membranes were considered alternative treatment options, vertical ridge augmentation was carried out to achieve an optimal implant to crown ratio, thereby minimizing open interproximal spaces, enhancing cleansability, and esthetics. Edentulous ridges were reconstructed in order to be leveled off with the adjacent periodontium of neighboring teeth, thus avoiding or minimizing negative bone remodeling.

A complicated compromised alveolar defect morphology often requires three-dimensional reconstructive surgery before or at the time of implant placement resulting in proper crestal bone levels with long-term stability around dental implants. The GBR procedure has high efficacy and predictability and is suitable for both horizontal and vertical ridge reconstructions [28]. However, the literature related to vertical GBR is very scarce, consisting of either prospective or retrospective case series.

The number of cases included in the present case series exceeds those reported in previous studies [16]. In the present study, 6 sites were treated by simultaneous GBR, while 18 sites received a staged approach. The majority of the patients were females, presumably with better compliance, with more health consciousness, and willing to undergo complex therapy. All cases were treated using the same split-thickness flap design, and all implants demonstrated successful osseointegration. Favorable peri-implant hard tissue surroundings were created around 36 out of 36 of the implants treated with the staged and around 7 out of 9 implants treated with the simultaneous approach.

Periosteal fenestration and vertical-releasing incisions are commonly used for vertical GBR to elevate a tensionless flap [11, 18, 29, 30]. Nevertheless, this flap design often results in complications such as swelling, bleeding, and patient discomfort, as well as flap perforation and graft exfoliation in 2.5–10% of the cases, depending on the augmentation technique [19]. One of the main causes behind these complications is probably the placement of deep periosteal incisions, which interrupts periosteal blood vessel circulation. Increased tissue swelling due to postoperative blood stasis generates tension at the crestal incision line which, in turn, may compromise wound healing and may lead to premature membrane exposure [20, 31].

The advantage of the present, prospectively evaluated surgical technique is the possibility of the bilaminar wound closure and the increased extensibility of the buccal mucosa, which will lead to a tension-free flap adaptation thus minimizing postoperative complications related to wound dehiscences. Uninterrupted blood supply induces optimal flap revascularization, which predictably results in uneventful early wound healing and moderate postoperative swelling and bleeding as well as membrane exposure. In the present study, only 1 out of 24 surgical sites (4.2%) demonstrated early membrane exposure, which is lower compared with that in literature [21, 25, 28, 32–35]. The only patient demonstrating early membrane exposure displayed one single tooth gap, where flap mobilization and tension-free wound closure are technically more challenging, compared with cases involving larger edentulous sites.

AP can be harvested either extraorally or intraorally and possesses substantial osteoinductive activity; however, it is prone to resorption. Particulate xenogeneic bone substitutes exhibit long-term volume stability and excellent osseoconductive capacity. In the present study, the application of a 1:1 mixture of AP and BDX was chosen, similarly to that described by Urban and co-workers [36]. This appears to represent a golden mean allowing for an optimal balance between graft remodeling and tissue stability. At reentry, clinically sufficient quality and quantity of newly formed hard tissues were observed in all cases. This is comparable with the results reported by several other authors applying 1:1 mixture of AP + BDX [24].

The titanium-reinforced non-resorbable e-PTFE membrane as a mechanical barrier is capable of preventing soft tissue migration and protecting the blood clot to achieve vertical hard tissue gain. The outer surface of the e-PTFE membrane has an open microstructure portion, while the inner surface is completely cell occlusive [37]. Former studies have demonstrated that the porous size determines regenerative capacity and clinical handling. Larger porous size could enhance biological effects; nevertheless, bone-membrane surface contact is considered to be too high, and therefore, membrane removal is difficult [38]. Bacterial colonization in case of membrane exposure could lead to bacterial infection after 4 weeks, which results in decreased hard tissue formation. In the present study, we have successfully used a new type of PTFE membrane for vertical GBR. The d-PTFE membrane has smaller pores; nevertheless, recent studies proved that the regenerative capacity is similar to e-PTFE membranes. d-PTFE is suggested for socket preservation, and staged and simultaneous vertical augmentation [22, 39]. In case of membrane exposure, the d-PTFE membrane temporarily inhibits biomaterial-centered bacterial adhesion and infection. Previous studies demonstrated successful socket preservation with d-PTFE membrane when left intentionally exposed [40, 41]. In our study, unexpected membrane exposure occurred only in one case of the simultaneous group. Six weeks later, the membrane was removed, and soft tissue ingrowth was observed underneath. A healing abutment was placed; the implant showed no considerable crestal bone loss at the time of loading.

The amount of newly formed hard tissues is critical to create a stable environment around implants for long-term success. The efficacy of augmentation procedures is directly related to the extent of the newly created peri-implant bony surroundings. Therefore, accurate standardized evaluation of vertical and horizontal hard tissue dimensions before and after augmentation procedures is necessary to judge treatment efficacy. With the recent development in 3D imaging and computer technology, pre- and postoperative linear dimensions and volumetric changes may be measured and visualized precisely. Still, the vast majority of data reported in literature is based on direct clinical measurements only. These assessments rely on the application of several types of probes or surgical calipers. Moreover, measurement inaccuracy due to limited visualization and positioning of the registration devices cannot be avoided intraoperatively. According to most of the relevant publications, vertical and horizontal gains following augmentation procedures are routinely registered at the utmost extent of reconstructed sites; however, this does not always represent actual implant positions. Due to the abovementioned inevitable difficulties, direct clinical measurements cannot be standardized over a large number of interventions. Therefore, we aimed at applying a standardized radiographic 3D evaluation approach, registering utmost linear and volumetric changes at actual implant positions in the present study. Cases treated by simultaneous GBR represented the only exception, since a second CBCT scan could not be accepted ethically according to the ALARA principles [42].

According to previous studies, 3.6–5.5-mm vertical hard tissue gain can be obtained after staged 3D GBR procedures utilizing different grafting materials. In 2003, Artzi and co-workers could reach 5.2-mm vertical hard tissue gain on average with titanium meshes and BDX [25]. In 2004, Roccuzzo and co-workers used AB covered by titanium meshes, and reported an average vertical gain of 4.8 mm [43]. In 2005, Proussaefs and Lozada’s vertical hard tissue gain was 4.8 mm 6 months after GBR, utilizing AP and AP + BDX without any barrier membranes [44]. Roccuzzo and co-workers used AB with and without titanium meshes. They observed a mean of 4.8-mm vertical hard tissue gain in the titanium mesh group, and a mean 3.6-mm new hard tissue with blocks alone [44]. In our study, 4.5 ± 2.2 mm was the average vertical hard tissue gain in the staged group, which compares well with the previous achievements despite utilizing AP + BDX only without bone blocks, covered by d-PTFE membranes. Urban and co-workers found 5.5-mm vertical hard tissue gain following the application of AP + BDX and d-PTFE membranes, nevertheless, with a conventional full-thickness flap design with vertical and periosteal releasing incisions [22].

According to the literature, 2.1–5-mm vertical hard tissue gain can be obtained after simultaneous 3D GBR procedures utilizing various grafting materials. In 1997, Corrente and co-workers utilized calcium carbonate and fibronectin sealing system around dental implants and observed a mean gain of 2.1 mm [45]. Simion and co-workers used e-PTFE membranes with AP or demineralized freeze-dried bone allograft (DFDBA) particles around implants. The AP group showed an average of 5 mm; the DFDBA group showed an average of 3.1-mm vertical hard tissue gain [12]. In 2007, Merli and co-workers applied e-PTFE membranes or resorbable collagen barriers with osteosynthesis plates for vertical augmentation at implant placement. The grafting material was AP. In the e-PTFE group, the mean vertical hard tissue gain was 2.5 mm, while in the resorbable barrier group, the amount of newly formed hard tissue measured 2.2 mm [34]. In our present study, we observed a mean 3.2 ± 1.9 mm vertical hard tissue gain by combining AP + BDX with d-PTFE membranes in the simultaneous group. Among previous reports, only Simion and co-workers showed higher vertical hard tissue fill (5 mm), nevertheless by utilizing AP only in combination with e-PTFE membranes.

The horizontal dimension of vertically augmented sites is crucial for long-term crestal bone stability. Based on the available data from the literature, a 1.5–2-mm facial bone width around dental implants is needed, which practically requires 7–8-mm crestal bone width in the horizontal dimension in cases of a standard, approximately 4-mm diameter implant [46–48]. Data reporting on the horizontal hard tissue gain following vertical GBR is scarce and are available only for horizontal GBR procedures, which cannot be compared with the results of the present study. Buser and co-workers measured the width of the alveolar ridge before and after horizontal GBR and reported an average horizontal hard tissue gain of 3.5 mm [49]. In 2008, Hämmerle and co-workers reported comparable outcomes, achieving 3.6-mm horizontal hard tissue gain [50]. Wallace and co-workers detected 4.6-mm horizontal hard tissue gain 6 months after horizontal augmentation with cancellous freeze-dried allograft bone blocks, as confirmed by CBCT radiographic evaluation [51]. Da Costa and co-workers applied allogenic bone blocks alone or impregnated with autogenous bone marrow during horizontal GBR. Their results have shown a mean horizontal gain of 2.2 mm and 4.6 mm, respectively [52]. In our study, the horizontal hard tissue gain 9 months after vertical GBR averaged 8.7 ± 2.3 mm after staged GBR procedures, and 6.5 ± 0.5 mm after simultaneous GBR procedures, respectively. From a clinical point of view, these results may ensure predictable crestal bone stability which is one of the important criteria for short- and long-term clinical success.

## Conclusions

Within their limits, the present results have shown that staged and simultaneous vertical reconstruction of deficient alveolar ridges by means of GBR with titanium-reinforced d-PTFE membranes combined with a bilaminar split-thickness flap design delivered predictable hard tissue formation as determined clinically and radiographically. The used surgical approach resulted in favorable wound healing, low patient morbidity, and low rate of membrane exposure.

## Electronic supplementary material

ESM 1 (JPG 384 kb)

ESM 2 (JPG 356 kb)

ESM 3 (JPG 534 kb)

ESM 4 (JPG 1263 kb)

ESM 5 (JPG 527 kb)

ESM 6 (JPG 485 kb)

ESM 7 (JPG 337 kb)

ESM 8 (JPG 671 kb)
