# Supplementary figures and images for: Vertical-guided bone regeneration with a titanium-reinforced d-PTFE membrane utilizing a novel split-thickness flap design: a prospective case series
Source: Clin Oral Investig. 2020 Oct 10;25(5):2969–80. doi: 10.1007/s00784-020-03617-6 (PMC8060182; doi:10.1007/s00784-020-03617-6)

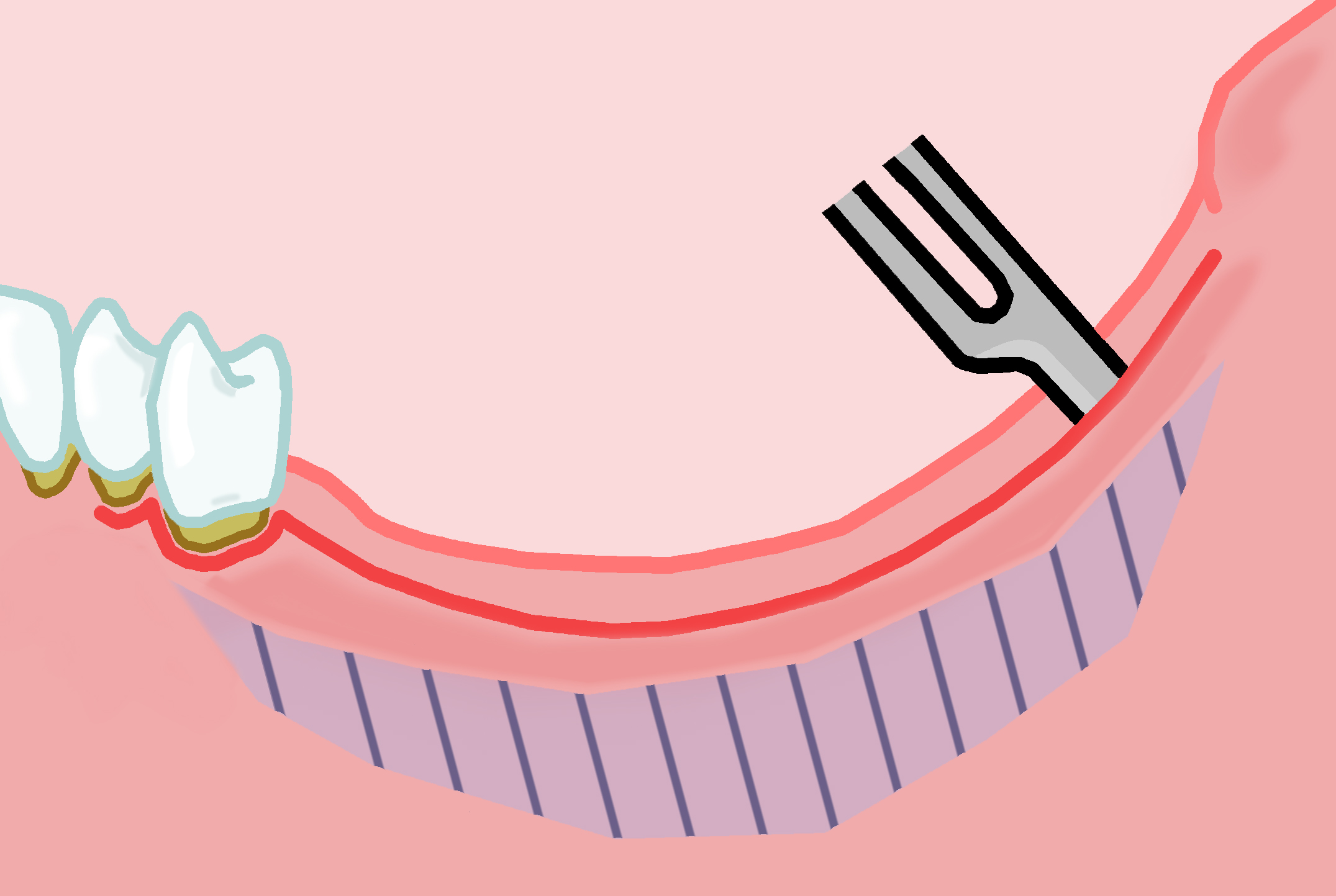

Supplement: Supplementary file 1 — (JPG 384 kb) [file 784_2020_3617_MOESM1_ESM.jpg]

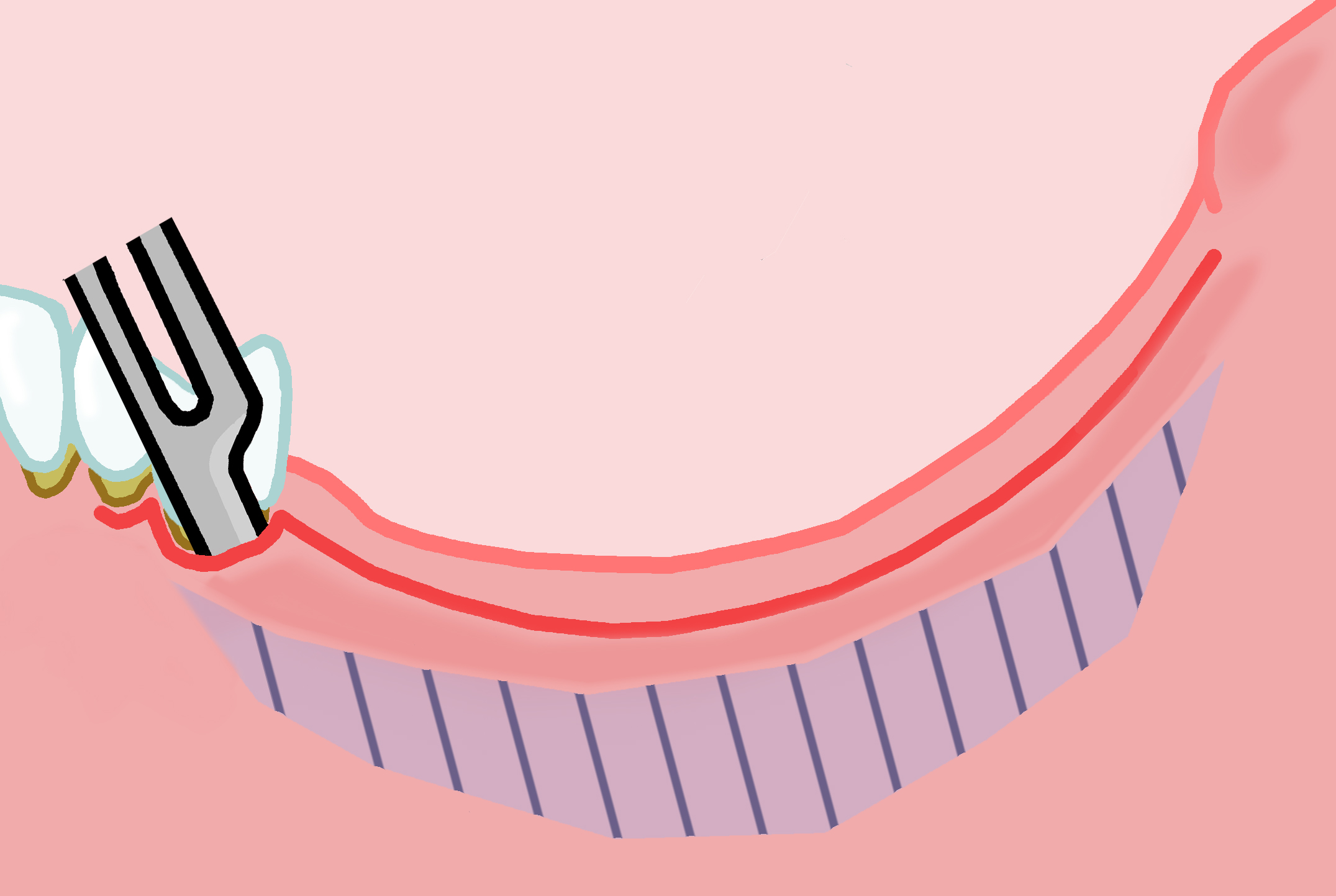

Supplement: Supplementary file 2 — (JPG 356 kb) [file 784_2020_3617_MOESM2_ESM.jpg]

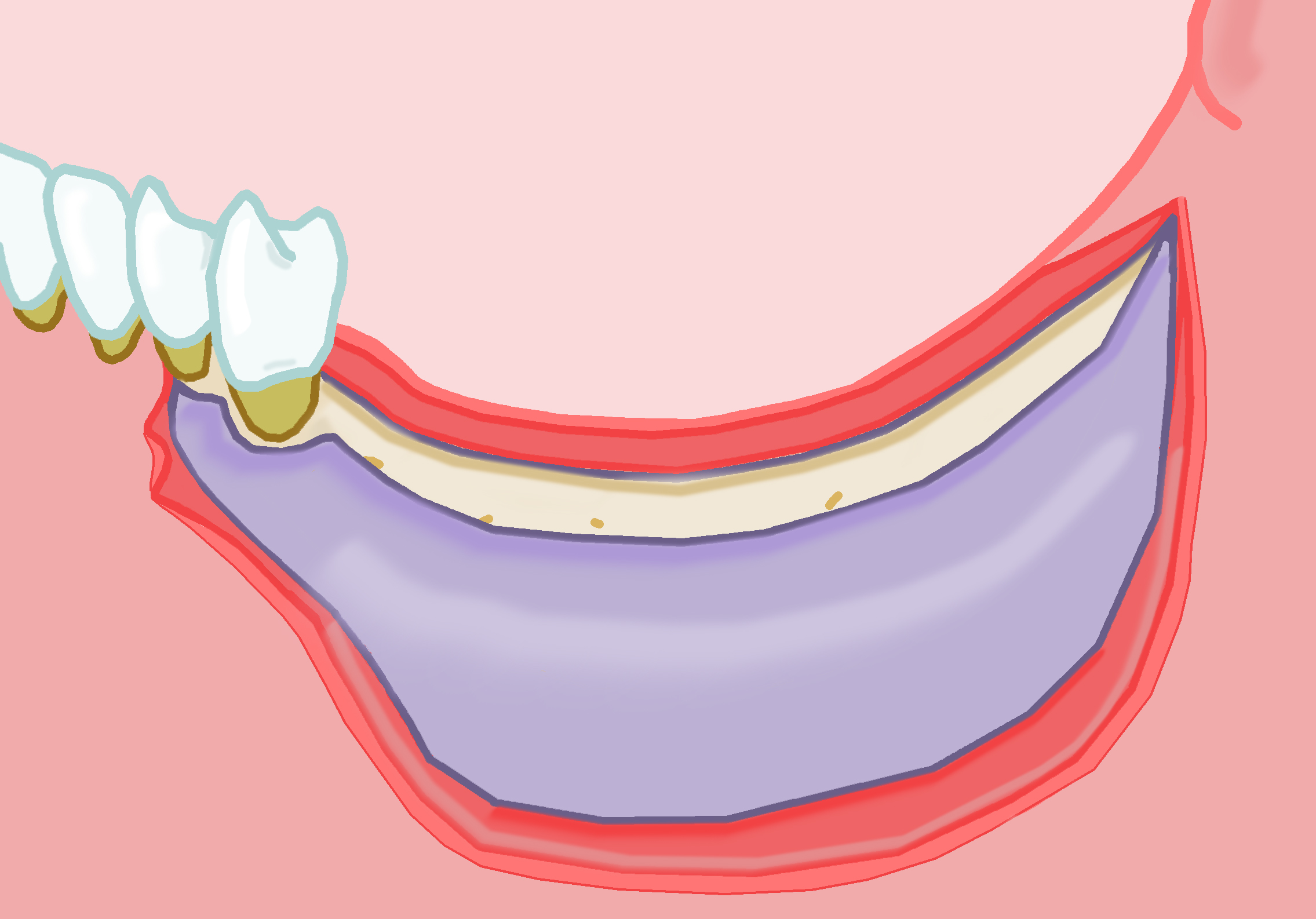

Supplement: Supplementary file 3 — (JPG 534 kb) [file 784_2020_3617_MOESM3_ESM.jpg]

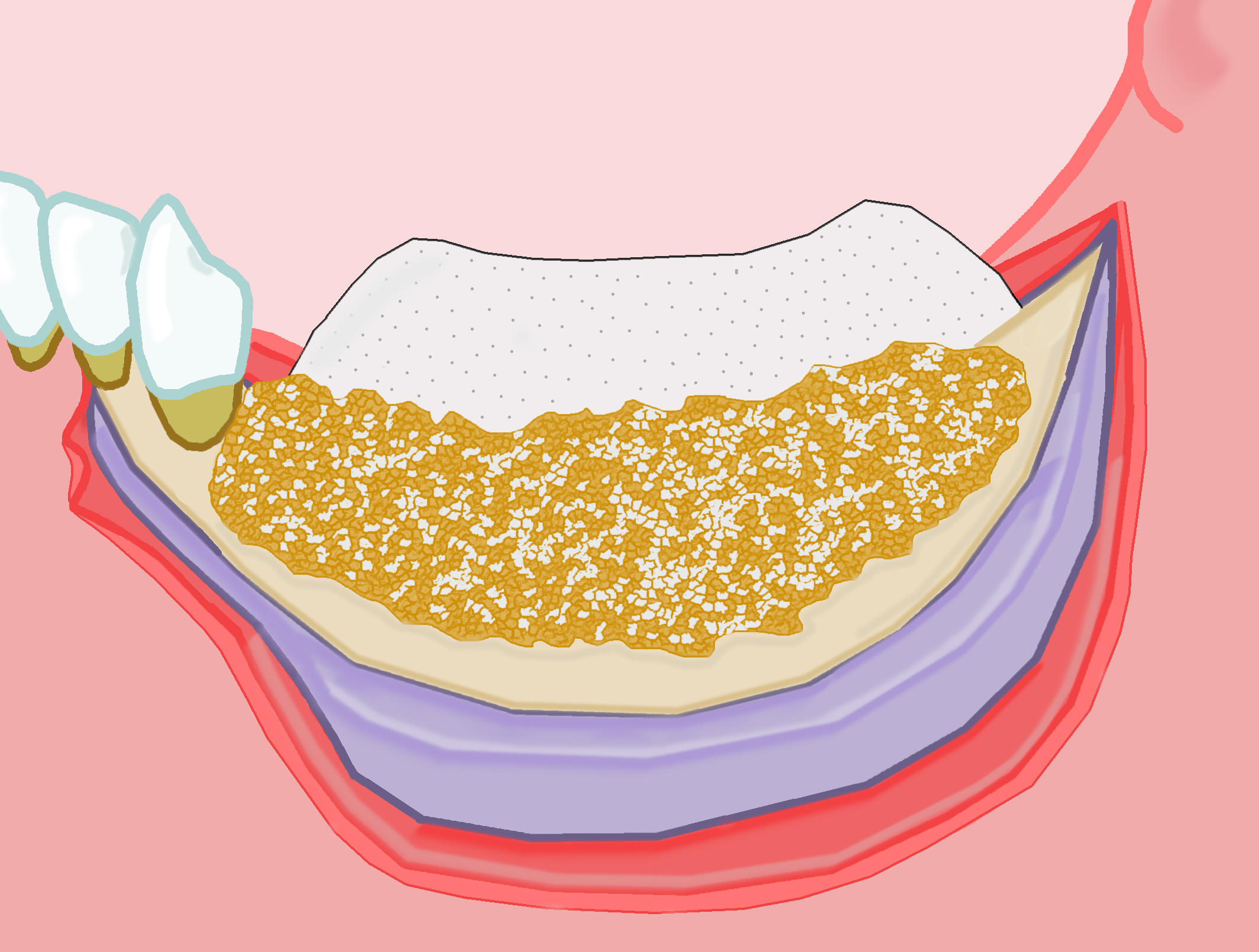

Supplement: Supplementary file 4 — (JPG 1263 kb) [file 784_2020_3617_MOESM4_ESM.jpg]

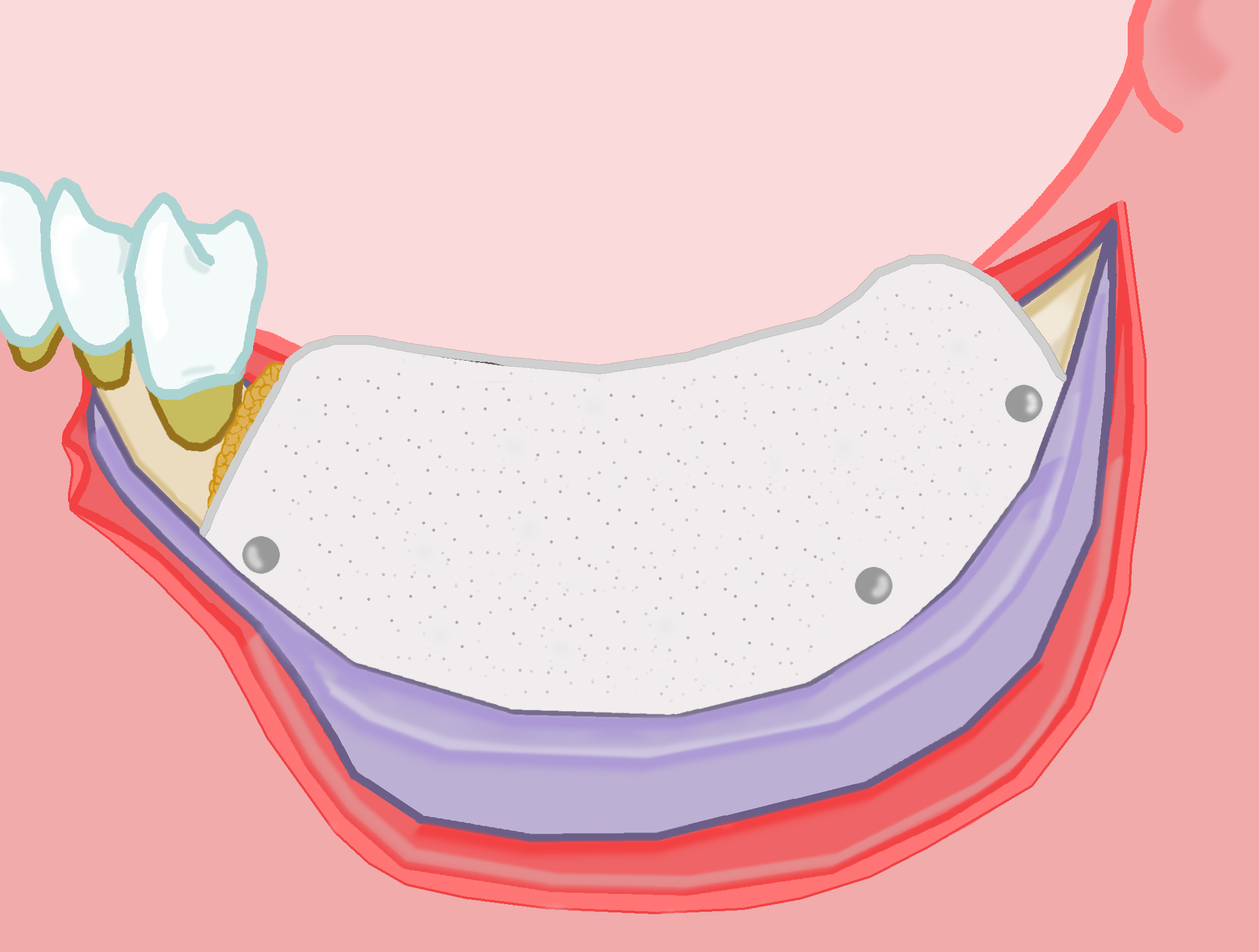

Supplement: Supplementary file 5 — (JPG 527 kb) [file 784_2020_3617_MOESM5_ESM.jpg]

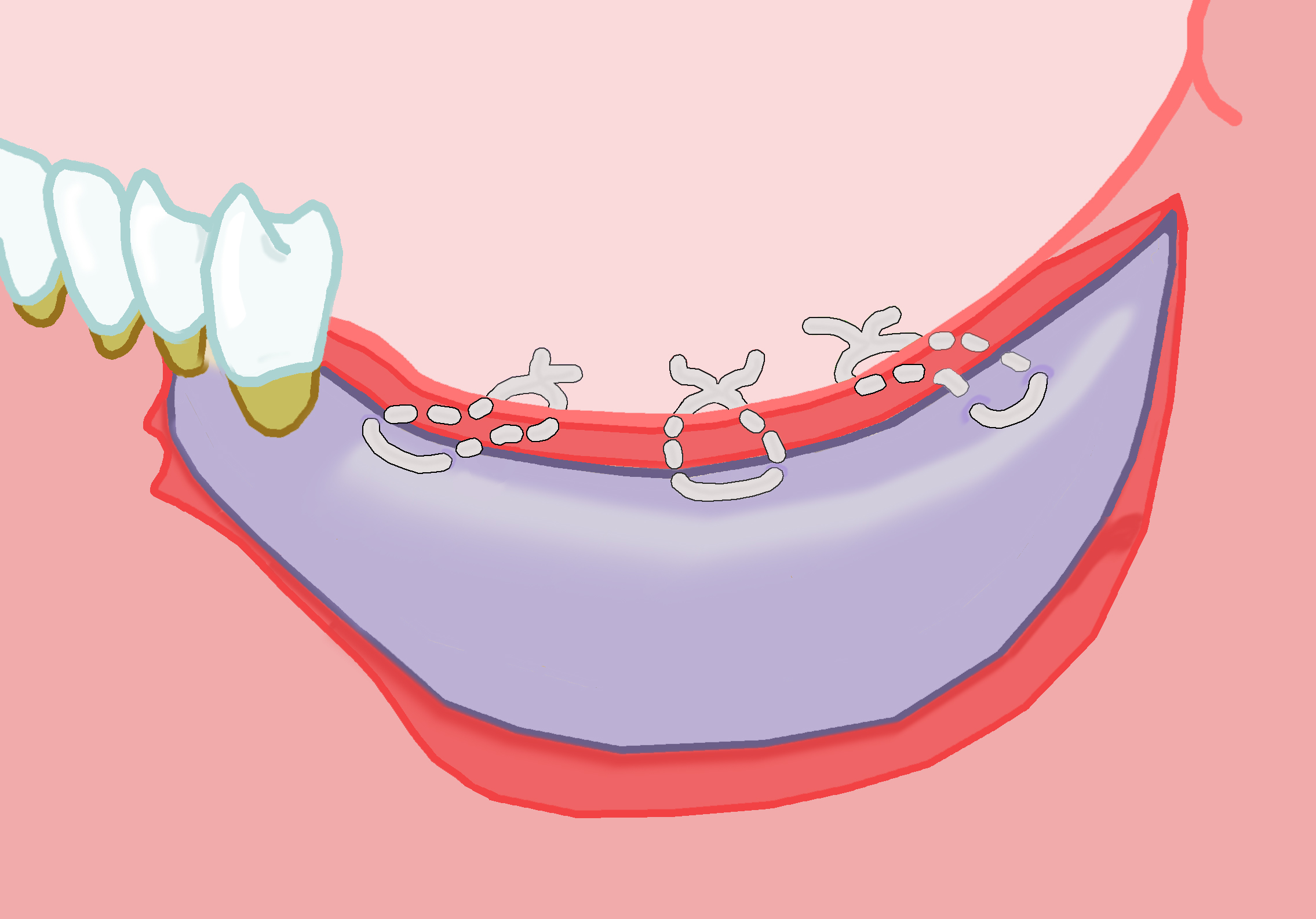

Supplement: Supplementary file 6 — (JPG 485 kb) [file 784_2020_3617_MOESM6_ESM.jpg]

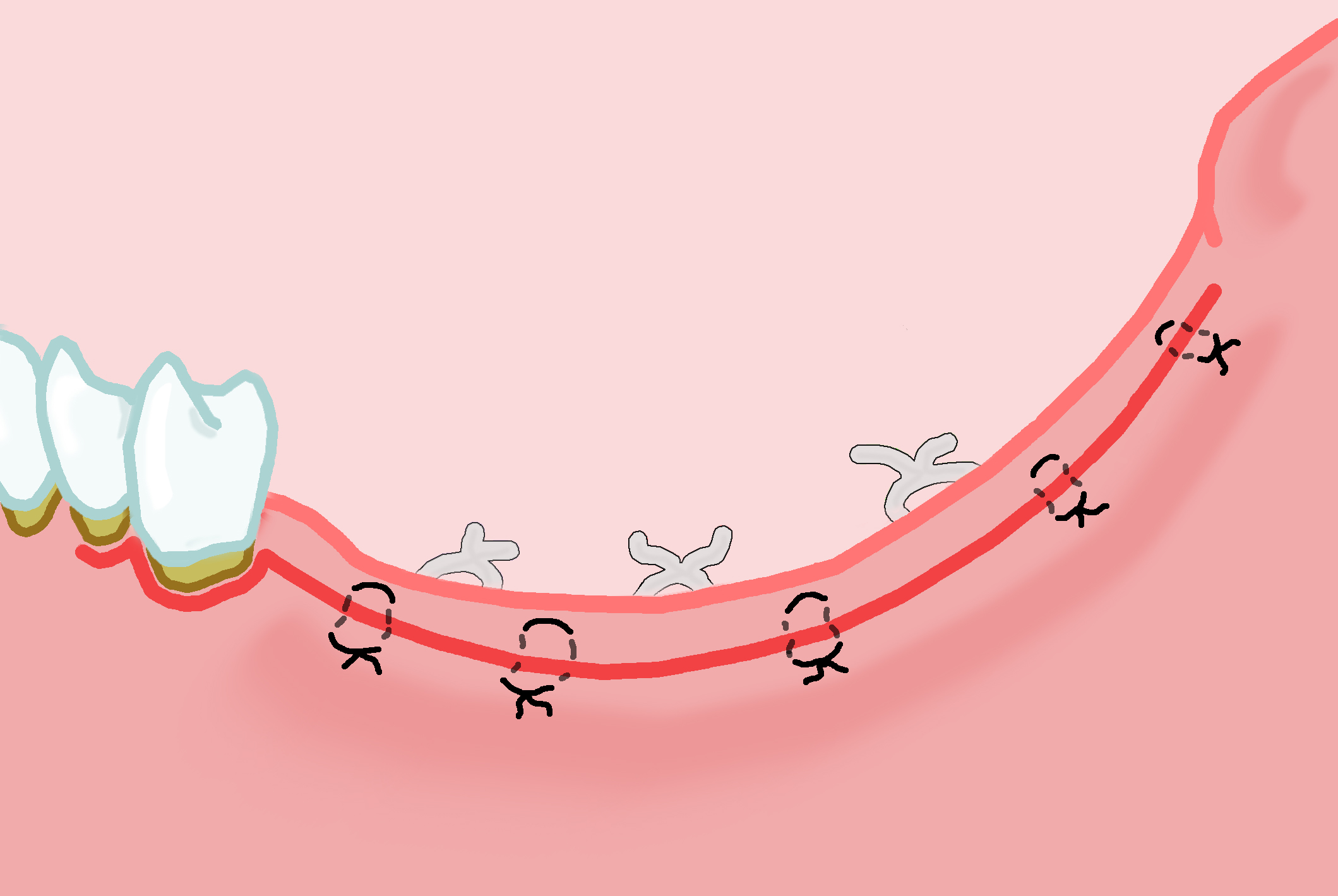

Supplement: Supplementary file 7 — (JPG 337 kb) [file 784_2020_3617_MOESM7_ESM.jpg]

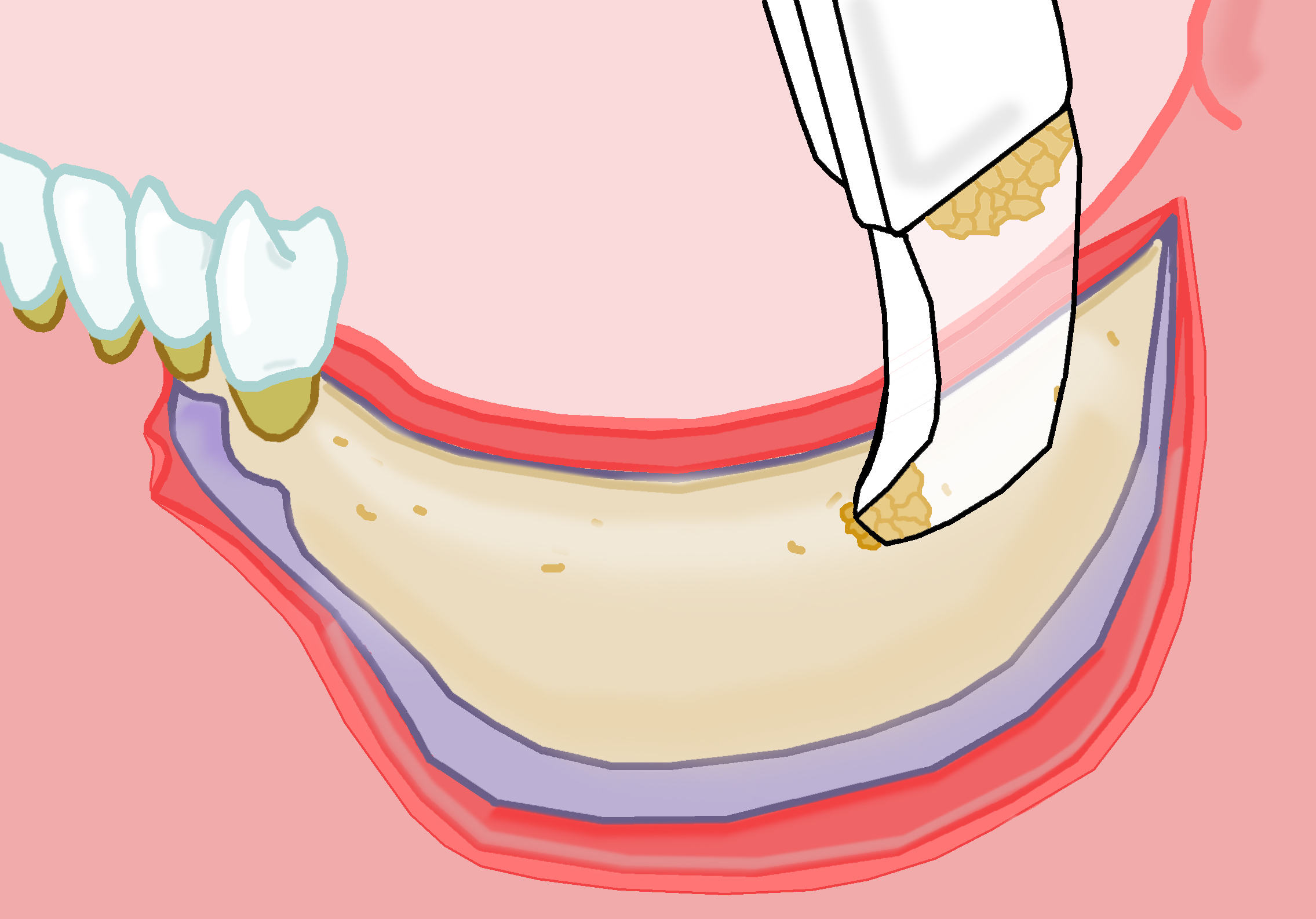

Supplement: Supplementary file 8 — (JPG 671 kb) [file 784_2020_3617_MOESM8_ESM.jpg]
